# Wearable Orofacial Technology and Orthodontics

**DOI:** 10.3390/dj11010024

**Published:** 2023-01-10

**Authors:** Sabarinath Prasad, Sivakumar Arunachalam, Thomas Boillat, Ahmed Ghoneima, Narayan Gandedkar, Samira Diar-Bakirly

**Affiliations:** 1 Department of Orthodontics, Hamdan Bin Mohammed College of Dental Medicine, Mohammed Bin Rashid University of Medicine and Health Sciences, Dubai 50505, United Arab Emirates; 2 Orthodontics and Dentofacial Orthopedics, School of Dentistry, International Medical University, Kuala Lumpur 57000, Malaysia; 3 Design Lab, College of Medicine, Mohammed Bin Rashid University of Medicine and Health Sciences, Dubai 50505, United Arab Emirates; 4 Discipline of Orthodontics & Paediatric Dentistry, School of Dentistry, University of Sydney, Sydney, NSW 2006, Australia

**Keywords:** wearable device, orthodontics, patient compliance, masticatory muscle, sleep disorders, smiling

## Abstract

Wearable technology to augment traditional approaches are increasingly being added to the arsenals of treatment providers. Wearable technology generally refers to electronic systems, devices, or sensors that are usually worn on or are in close proximity to the human body. Wearables may be stand-alone or integrated into materials that are worn on the body. What sets medical wearables apart from other systems is their ability to collect, store, and relay information regarding an individual’s current body status to other devices operating on compatible networks in naturalistic settings. The last decade has witnessed a steady increase in the use of wearables specific to the orofacial region. Applications range from supplementing diagnosis, tracking treatment progress, monitoring patient compliance, and better understanding the jaw’s functional and parafunctional activities. Orofacial wearable devices may be unimodal or incorporate multiple sensing modalities. The objective data collected continuously, in real time, in naturalistic settings using these orofacial wearables provide opportunities to formulate accurate and personalized treatment strategies. In the not-too-distant future, it is anticipated that information about an individual’s current oral health status may provide patient-centric personalized care to prevent, diagnose, and treat oral diseases, with wearables playing a key role. In this review, we examine the progress achieved, summarize applications of orthodontic relevance and examine the future potential of orofacial wearables.

## 1. Introduction

Considered by many as the first wearable technology, the pedometer was invented in the mid-1600s to quantify a person’s number of steps [1]. At the time, the main purpose of the device was to obtain a more objective number of steps, compared to a subjective approximation. Gradually, over time, improvements in computing and the miniaturization of electronic components led to a progressive reduction in the size of devices. Many of these innovative devices have sustained Moore’s law, an observation that the number of transistors in dense integrated circuits doubles about every two years [2]. The miniaturization of components combined with improved computational capacity with a peak rate of change in the 1990s [3] led to significant improvements. Advances in the field of data transmission also happened simultaneously, and a game changer was the evolution of wireless technology for exchanging data over short distances with the arrival of Bluetooth in the 1990s. Further improvements led to Bluetooth Low Energy (BLE), a wireless personal area network technology aimed at novel applications in healthcare, without compromising power requirements [4]. 

**Figure 1:**
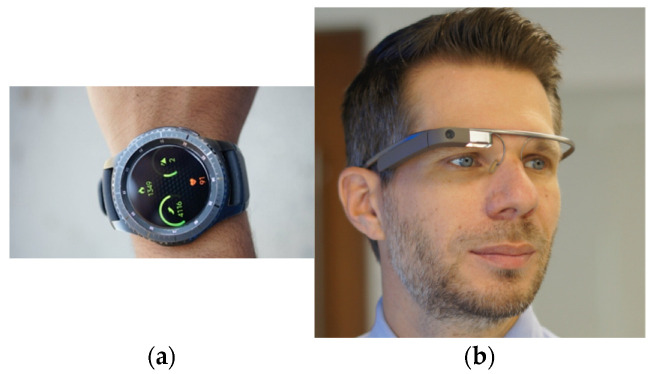
On-body wearables: (**a**) Physical activity and heart health tracker worn on the wrist; (**b**) Optical head-mounted smart glass.

From a single and mechanical modality, medical wearables have now evolved into mini-computers that offer multiple functions including the monitoring of heart rate, oxygen saturation, sleep, physical activity, body temperature, and blood pressure, to name a few. Not only has the connectivity of wearables to smartphones facilitated the access to information, it also has allowed simpler and smaller product designs. From the wrist, wearables have moved to other parts of the body such as the fingers, eyes, ears, areas of the face, and the head [5] (Figure 1). 

In a recent US national survey, 30% of the respondents said they use a wearable to collect health data on a daily basis [6]. Though the accuracy of some of the measurements collected via wearables is sometimes debatable [7,8], the real challenge for clinicians and hospitals is to access and leverage these data. While protecting the confidentiality and privacy of patients is often the first concern, a lack of system interoperability and connectivity, as well as data overload, prevents hospitals and clinics from using the large amount of data that wearables collect [9]. Despite this, during the Coronavirus pandemic, healthcare institutions around the world invested in platforms to integrate wearables owing to their capacity for continuous home-based health monitoring [10,11].

In the early 2000s, wearables drew the attention of scientists, who conducted multiple studies to evaluate whether these devices could positively impact people’s quality of life. Researchers rightfully hypothesized that knowing the current number of steps would encourage walking more [12]. Though the topic of whether or not wearables have a positive impact on people’s level of physical activity is still ongoing, research has demonstrated the usefulness of wearables in many different scenarios. For instance, in one of the largest clinical trials involving 400,000 participants, researchers showed the ability of an activity tracker to detect atrial fibrillation and thus potentially save hundreds of lives every year [13]. Wearables have also been used to monitor cancer patients during treatment [14], support the management of chronic stress [15] and as eye-wear to help patients suffering from autism [16,17]. 

This paper provides a review of wearable devices specific to the orofacial region and important from an orthodontic perspective. Wearable technologies for continuous, unobtrusive, and objective monitoring of compliance, sleep disorders, and jaw function and parafunction are first discussed. The paper then continues with the use of wearable technologies in orthodontic research, briefly describes risk assessment and regulatory aspects of wearables, and ends with a look at the future of wearable devices.

## 2. Compliance Monitoring

Patient compliance is crucial for the success of orthodontic treatment [18], and noncompliance during treatment can negatively influence the duration of treatment [19]. Although difficult to predict [20], patient-related factors that affect compliance include age, sex, and an understanding of the importance of wearing the appliances/retainers [21]. Reasons linked to poor patient compliance can be traced to pain, discomfort, and forgetfulness [22,23]. Presently, smartphone apps are available that can be used to send reminders to improve patient compliance [24,25,26], and with improved compliance, orthodontic treatment duration decreases [27].

In a clinical setting, the evaluation of treatment progress is considered representative of compliance. However, assessments for compliance conducted by the orthodontist and clinical staff could be biased based on their interaction with and impression of the patient [28]. Patient self-report is also often used for the assessment of compliance during [28] and after [22,29] orthodontic treatment. Self-report is widely used as it is practical, but it is prone to recall bias and often overestimates wear time [30]. Failure to detect a lack of compliance makes it challenging for the practitioner to ensure the effects of appliances have been reflected or results of treatment maintained. Hence, objective and reliable methods to evaluate compliance are important. 

Since the 1970s, there have been attempts to objectively measure compliance in orthodontics. Most early attempts were focused on monitoring compliance with head gear wear, probably due to heavy reliance on patient compliance for success and its popularity during the period [31]. On the other hand, a similar development of intra-oral devices for monitoring did not happen in the same period, possibly due to size limitations and lack of technology for safe functioning in moist environments. 

One of the earliest studies for monitoring headgear wear time used a miniature electronic watch with a memory circuit. The timer consisted of two switches that were activated when the appliance was worn and accumulated wear time until the appliance was removed. The study found that when patients were informed that they were being monitored, hear gear wear-time more than doubled [32]. In the late 1970s, using an Aledyne timer, younger patients were found to wear head gear for longer durations [33]. Several different designs of head gear wear-time monitors were published in the 1980s and 1990s [34,35,36,37,38]. However, most were bulky and obtrusive, offered only a cumulative measure of wear time, and were uncomfortable as electronic components needed to be placed in the neck strap. Interestingly, a consistent finding observed in most studies using wearable timers to measure compliance was that when subjects knew about being monitored, they were more inclined to wear the head gear [36,39,40]. 

**Figure 2:**
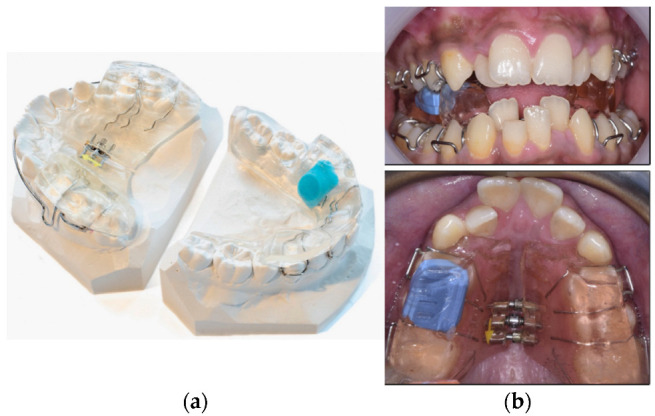
Micro-electronic thermal biosensors embedded in different locations of myofunctional appliances to monitor wear-time: (**a**) lingual flange of the lower block, Reprinted with permission from [45]; (**b**) in the upper block, Reprinted from [46], with permission from Elsevier.

Although progress in the widespread adoption of wearable technology for compliance monitoring has been slow, the role of technology as a strategy for creating a compliant patient was emphasized in the late 1990s [41]. Over the years, micro-electronic sensors for monitoring compliance have been successfully embedded in functional appliances [42,43,44,45,46] (Figure 2), appliances for sleep disorders [47,48,49,50], facemasks [51,52], and active removable appliances [44,53,54,55] (Table 1). Long-term removable retainer wear is recommended post-orthodontic treatment and microsensor-embedded retainers have been used to overcome the practical challenges associated with monitoring compliance [54,56,57,58,59] (Table 1). 

The majority of the sensors used intraorally are temperature-sensitive microsensors that detect and measure intraoral temperature when the appliances are in the mouth and translate the information into wear time [45,49,50,53,54,58,59,60]. The safety and cytotoxicity of thermal microsensors have been successfully evaluated [61], reinforcing that it is possible to opt for them to objectively monitor compliance. In addition to measuring temperature to a 0.1 °C resolution, a micro-electronic sensor that also detects the spatial orientation/movement in the x-, y-, and z-axes to a 2° angulation resolution and head position (DentiTrac^®^, Braebon Medical Corporation, Ottawa, ON, Canada) has also been described. Dedicated software (Braebon Medical Corporation, Ottawa, ON, Canada) is used to convert data recorded by the sensor into wear time [47,48,62]. Data from intra-oral micro-electronic sensors are read using dedicated stations or may be transferred to a smartphone via Bluetooth in real-time [63]. 

Although objective monitoring with wearable microsensors offers acceptable accuracy with mean under-reporting of 4% [64], there are still drawbacks. The area where the sensors are embedded within the appliance is reported to affect wear-time measurements, with sensors located in the lower buccal sulcus recording a wear time closer to the actual wear time compared to sensors located in the palate [64]. Embedding sensors also adds to the treatment cost and the bulk of appliances, which may in turn deter wear [65]. All micro-electronic sensors used today incorporate batteries, which limit life span, and they also have limited data storage capacity, thereby limiting monitoring periods, which often last only a few months [66,67]. In addition, micro-electronic timers can become detached and may be prone to loss of data and premature deactivation. For successful future applications, micro-electronic sensors with further reductions in size, longer battery life, and improvements in reliability are still needed [68]. 

Success with clear aligner treatment also relies heavily on compliance with up to 22 h of wear time recommended on a daily basis. Remote monitoring has been found to be useful in assessing compliance with clear aligners [69,70]. Although not an objective monitoring method, clear aligners embedded with a food-grade dye (erioglaucine disodium salt) that dissolves when exposed to saliva are an available option for compliance monitoring. A subjective evaluation of color change (five shades from dark blue to clear) is used to obtain a visual representation of wear time [71,72]. However, not much is known about whether these colorimetric compliance indicators are affected by intraoral pH and temperature. 

Similar to extraoral monitoring, patients have mostly affirmed better adherence to wearing intra-oral appliances when they were aware that their compliance was measured [56], although there is evidence suggesting that awareness of being monitored with micro-electronic sensors did not improve compliance [54]. Patients have also reported positive feelings when they were able to graphically view information about wear duration in real time [63]. An objective understanding of compliance permits setting appointment schedules, keeping in mind individual treatment needs, helping motivate patients, and also eliminating inconsistencies arising from clinical judgments or subjective self-reports. It also needs to be remembered that objective monitoring of wear time is only one among the several factors that influence patient compliance [73].

## 3. Sleep Measurements

Both subjective (self-report) and objective (physiological monitoring) approaches can be used to measure sleep quantity (duration) or sleep quality (sleep architecture and pathology). Discrepancies of over-estimation (typical of healthy adults) and under-estimation (typical in chronic insomnia) between objective and subjective measures have also been reported [77]. 

Orthodontists are well positioned to perform obstructive sleep apnea (OSA) screening assessments and refer at-risk patients for a diagnostic evaluation [78]. Overnight polysomnography (PSG) in a sleep laboratory is considered the gold standard in the diagnosis of OSA. However, access to PSG might not be widely available due to the scarcity of sleep laboratories and sleep technologists, in addition to the high cost of the in-laboratory studies that may not be covered by insurance. Overnight recordings similar to PSG can also be performed at home using polygraphic home sleep apnea tests (HSATs), which use a restricted number of measured signals and may be less costly and more efficient in some populations [79]. Interestingly, during the Coronavirus pandemic, HSATs were recommended and preferred over in-lab studies for OSA diagnosis by some sleep organizations [80]. Both PSG and HSAT trade diagnostic accuracy with obtrusiveness. They are unsuitable for performing population screening and monitoring OSA variability across multiple nights. 

Wearable devices could provide a level of unobtrusiveness unachievable with standard techniques and enable faster OSA screening with improved long-term characterization over multiple nights. Newer multi-sensor sleep wearables integrate additional metrics such as heart rate, skin conductance, skin temperature, blood oxygenation, accelerometer data [81], and mandibular motion [82].

Once the diagnosis of OSA is confirmed, the use of procedures or oral appliances (OA) in appropriately selected patients is the next step. First-line therapy for moderate to severe OSA is continuous positive airway pressure (CPAP), which prevents upper airway collapse during sleep. However, only some patients [83] remain compliant with treatment for an extended period due to reasons rooted in the OSA severity, obtrusiveness of the treatments, and lack of perceived beneficial effects [84]. A recent randomized controlled clinical trial of a machine learning–based intelligent monitoring system (MiSAOS intelligent monitoring) aiming to improve CPAP compliance in patients with OSA reported excellent patient satisfaction and proved cost-effective [85].

OAs, which include mandibular advancement appliances (MADs), are another effective option for OSA management in appropriately selected patients. For OA, in addition to proper checking and fitting and the need for titration, the evaluation of compliance is important [78]. Microsensors have also been embedded in OA to monitor compliance in OSA subjects. The earliest study used a miniaturized, temperature-sensitive, embedded monitor. This ceramic monitor had a memory system and temperature sensor to monitor wear-time based on a temperature measuring above 31 °C. The damaging effect of saliva, heat intolerance of the electronic components, and energy consumption over a long period were problems with this early attempt [86]. However, no adverse effects were reported in a later study that embedded a different temperature-sensitive microsensor in a MAD to objectively monitor compliance [49]. Recently, long-term monitoring using a temperature-sensitive microsensor showed relatively high objective OA use on 1-year follow-up [50]. Currently, three microsensors that can be used to monitor removable OA use for OSA are available commercially. The sensors differ in terms of data-recording, life span, signal readout, and storage capacity. The accuracy of these temperature-sensitive microsensors was tested in an in vitro study [87] that found the TheraMon (MC Technology GmbH, Hargelsberg, Austria) microsensor to be accurate in both short- and long-term durations. In comparison, the AIR AID SLEEP sensor (AIR AID GmbH & Co. KG, Frankfurt, Germany) and the DentiTrac microsensor (Braebon Medical Corporation, Ottawa, ON, Canada) significantly underestimated and overestimated wear time, respectively. 

In a recent survey, 12% of researchers interested in real-world sleep outside a controlled laboratory environment preferred wearable devices that are worn on the head for sleep measurements [88]. Other locations for orofacial wearables focusing on sleep measurements include the chin, earlobe, and over the eyes as a sleeping mask. The wearable sleeping mask (Neuroon) combines advanced brainwave- and pulse-measurement technologies to analyze and improve sleep quality. The earlobe wearable (Lumafit) detects heart rate and sends it to a smartphone for viewing, analyzing, and subsequently guiding the user to a state of deeper relaxation to supposedly teach a form of breathing to lower stress, increase focus, and improve sleep quality. Recently, a commercial system (Sunrise System) consisting of a miniature coin-sized wearable sensor embedded with an inertial measurement unit (IMU) that is worn on the chin [84] was described as showing potential for diagnosis of OSA [89,90,91]. 

## 4. Jaw Function

Oral behaviors are frequently assessed based on questionnaires [92,93] or occasionally in real time [94,95]. The simplicity and affordability of questionnaires make them well-suited for use with large samples, but they are susceptible to recall bias, thereby limiting external validity. Repeated on-site measurements from participants, based on real-time experiences, depend on the careful timing of assessments and compliance of participants in responding to prompts, and substantial time and effort are required by participants in being actively engaged with the measurement instrument. 

The importance of combining objective with subjective measures when evaluating masticatory function has been previously emphasized [96]. Historically, instrumental approaches to objectively study jaw function have included measurements of occlusal bite force, jaw kinematics, and electrical energy in the masticatory muscles [97], with occasional integration of multiple measurements [98,99,100,101,102]. Coupling measurements of masticatory muscle electromyography (EMG) with those from jaw motion tracking provides information about the correlation between jaw movements and masticatory muscle activity (MMA), thereby improving our understanding of orofacial function [100,103,104,105,106] and parafunction [107,108,109,110]. Advances in technology and miniaturization permit instrumental approaches to understanding oral function [111,112] and parafunction [113,114,115] in naturalistic settings.

In comparison with intramuscular EMG, surface electromyography (sEMG) is non-invasive, seemingly simple to apply, and can provide real-time information about muscle activations and is therefore well suited for understanding muscle activity in the natural environment. Masticatory muscle sEMG provides objective, valid, and reproducible data about muscle contractions [116] that has enhanced our understanding of masticatory muscle function [117] and dysfunction [118]. Of the muscles used for mastication, the masseter muscle is easily accessible due to its size and superficial position and is therefore the most extensively investigated using sEMG [119,120]. Data on masticatory muscle contractions (frequency, intensity, duration) and oral behaviors can be acquired using sEMG over extended periods of time. 

With instrument-based approaches, real-time sEMG is the theoretical reference standard for understanding long-term oral parafunctional behaviors [121] and for studying awake bruxism [122]. sEMG equipment can be categorized as stationary, portable (wired or wireless), or wearable [123]. In comparison to stationary lab- or hospital-based equipment [124], fewer studies have used portable equipment or wearable devices. 

### 4.1. Masticatory Muscle sEMG

Portable sEMG equipment has been extensively used in sleep bruxism (SB) research, possibly due to the reduced amount of body movement that occurs during sleep in comparison to when awake. Despite the wired and obtrusive nature of portable equipment, their use has continued over time since the restricted motion during sleep did not affect the quality of recordings. Until recently, data on objectively recorded masticatory muscle sEMG during daytime in the natural environment were limited, largely due to the dearth of wireless recording equipment that can be used unobtrusively in real-life settings. Wearable sEMG devices worn on the body without being obtrusive to daily activities are presently available for long-term recording of MMA.

A wearable single-component, single-use, disposal electromyographic device (BiteStrip) designed to screen masseter EMG was first described in 2007. The device was successfully validated against traditional masseter EMG measurements with acceptable sensitivity and positive predictive values [125]. The wearable device was found to be a moderately accurate screening tool for the diagnosis of SB [126] and was successfully used in a clinical setting to evaluate the effect of a MAD in SB subjects [127].

In the same year, a wired sEMG device was described consisting of a display and two recording channels, with data being transmitted to a computer. The device was used to record masseter EMG activity during sleep to study the effect of an oral appliance used to relieve temporomandibular disorder (TMD) symptoms [128]. In the following year, an advanced wearable version of the same device placed around the forehead, with electrodes near the temporalis muscle and featuring a biofeedback function, was produced. This wearable device was designed to record EMG, process signals to detect a particular activity (e.g., tooth grinding or tooth clenching), and provide battery-powered electrical stimulation as biofeedback [128]. This wearable device was used in conjunction with PSG in subjects with self-reported SB and did not affect sleep parameters when contingent electrical stimulation was at non-painful intensities [129]. New and improved versions of this ambulatory device were later proposed as a valid option in SB assessment [130] with a low to modest diagnostic validity [131]. Interestingly, in children, MMAs using the device and from PSG assessment were not correlated as far as the number of muscle contraction episodes were concerned [132]. However, a reduction in the level of nocturnal parafunctional activity bringing about symptomatic improvement was possible using this wearable device [133]. 

In 2011, a wearable single-channel recording system (EMG-BF) hidden behind the ear, akin to a hearing aid, was described with electrodes lying over the anterior temporalis muscle. This wearable device had reduced visibility, permitting sEMG measurements without interfering with daily life. Acceptable agreement was found between self-reported data and objective MMA measurements with this wearable device, suggesting that self-reported daytime clenching is a reliable screening parameter for awake bruxism [134]. Using this device, daytime clenching was shown to be associated with a tendency towards anxiety with 3.5 times greater total muscle activity in the clenching group compared to the non-clenchers [135]. The same device combined with audio-based biofeedback was found to be useful in reducing daytime clenching in the short-term in natural conditions [136]. 

A compact wearable sEMG device (Bruxoff) using unique bipolar concentric electrodes was used in conjunction with electrocardiography (ECG) measurements in subjects with SB. Conjoint analysis of the wearable sEMG along with ECG was found to be useful in supporting a clinical diagnosis of bruxism [137]. Good reproducibility of SB measurements over time was seen with the device [138]. However, instrumental SB diagnosis performed with this EMG/ECG device did not correlate with clinical assessment [139]. The concentric electrodes used with the device being isotropic have the advantage of being invariant to rotations while being positioned over the muscle of interest. Concentric electrodes were also used with another single-channel wearable sEMG recorder suitable for long-term use in the natural environment [140].

In 2014, a custom wearable system incorporating analog signal processing, differential amplification, and a wired data logger was used to understand the relationship between diurnal bruxism and the progression of tooth loss. Using this system, excessive wake time masseter sEMG activity was found to be more destructive for dentition than sleep time activity [141]. The same equipment found that tonic episodes (continuous EMG activity of at least 2 s duration with intensities above twice the baseline noise level) correlated with facial pain, and 7.5–25% maximum voluntary contraction (MVC) was suggested as a range for future clenching studies [142]. Discriminating low-level clenching from speech is important, especially during wakefulness, and a reliable wearable system was successfully able to evaluate low-level masseter EMG activity related to low-level tooth clenching while discriminating speech activity [143]. A wearable sEMG device (Actiwave) capable of measuring MMA events during the daytime with high stability and reliability under normal living environments has also been described [144]. 

**Figure 3:**
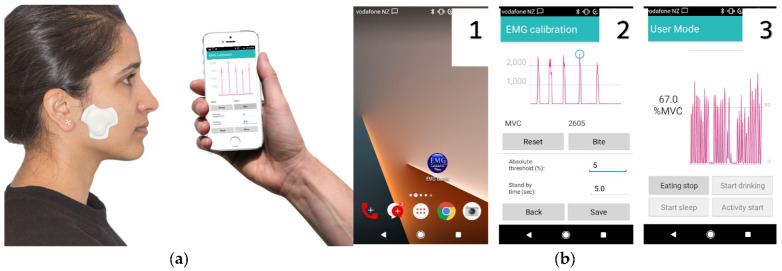
Wearable sEMG device for MMA monitoring: (**a**) an android smartphone is used for logging and displaying muscle activity in real-time; (**b**) a dedicated smartphone app (1) helps in initial calibration (2) and recording of MMA that is displayed graphically on the screen in real-time. Note the multiple rhythmic medium amplitude spikes characteristic of chewing activity (3).

Recently, an ultra-miniature sEMG wearable (FLA) [145] was found to be valid for the diagnosis of SB when a cut-off value appropriate for single-channel EMG is used [146]. Commercially available wireless wearable sEMG systems have also been used for lab-based assessment of masseter muscle activity [147,148]. Recently, the successful development and validation of a novel wearable sEMG device (Figure 3), which uses a smartphone for logging and real-time graphical representation of MMA in real-life settings, was reported [149]. This smartphone-assisted wearable sEMG device has been used successfully in children for home-based diet monitoring [150,151], in women to study correlations between MMA and physical activity [152], and in women with masticatory muscle pain to study wake-time masseter activity [153]. 

### 4.2. Tracking Mandibular Motion (TMM)

Recording of mandibular motion has been used to understand the normal jaw function and for the diagnosis and treatment of TMDs. Although the masticatory system is dynamic, methods used often relied on recording statistical points or single positions of the mandible (e.g., protrusion, lateral excursion, etc.). 

Devices and methods for TMM in the past have greatly improved our understanding of the masticatory system and mandibular movements. Historically, mandibular motion has been investigated using different methods and devices, often reflecting technology popular at the time. These have included mechanical [154], photographic [155], electromechanical [156], photoelectrical [157], cinephotographic [158], cineradiographic [159], ultrasound [160], electromagnetic [161], and magnetic [162] systems. Early graphic methods included tracing devices, which used “clutches” (a means for attaching the recording device to the patient’s jaws) attached to the teeth. They were capable of tracing the movement path in two dimensions but were unable to simultaneously time the movements. Interference of mechanical tracking devices with normal mandibular function was a common problem [163]. Photography and cinematographic techniques later eliminated the need for the cumbersome “clutches” and allowed the calculation of the timing of jaw movement; however, they were still limited to recording single-plane movement data [163]. A magnetometry approach was later introduced to sense changes in the magnetic field resulting from the movement of a permanent magnet attached to the lower incisors, permitting reliable quantitative and reproducible data of TMM in three dimensions [164].

Newer systems for tracking motion use measurements from a range of miniature sensors (mechanical, inertial, acoustic, magnetic, optical, or radio frequency) of which optical [165,166] inertial [167] and magnetic [82] have been successfully employed for TMM. As occlusion-free measurements are possible with magnetometry-based approaches, they are often used for TMM in lab-based [168], hospital-based [169], home-based [170], or ambulatory subjects [101,106,171].

Wearable devices that are lightweight and unobtrusive are essential for TMM in naturalistic settings. A simple system using accelerometers and a Hall-effect device temporarily glued to the upper and lower anterior teeth was used for recording vertical movements of the mandible in ambulatory subjects [171]. The accelerometer signals were integrated to give the position and movements of the mandible relative to the maxilla. Although the (relative) velocity and position records derived in this way were linear, they were found to drift when the jaw was stationary. Two permanent NdFeB magnets and elements of a two-axial fluxgate sensor array were used in a magnetometry-based jaw-tracking system in which the magnets were attached to a portion of the head and front tooth [172]. The proposed system was applicable for five degrees of freedom and achieved a positional accuracy within 2 mm. 

A miniaturized wireless inertial measurement unit (WB-3) consisting of a three-axis gyroscope, three-axis accelerometer, and three-axis magnetometer, which could measure acceleration, angular speed of jaw movement, and mouth-opening angle, was proposed for measuring jaw motion [173]. The updated version, WB-4 used nine-axis inertial sensors (miniaturized accelerometer, gyroscope, and magnetometer) to measure jaw motion [167]. The reduced weight and size of WB-4 along the Bluetooth module for wireless communication permitted it to be easily attached to the mandible without any physical restriction. 

Recently, a commercial system (Sunrise System) was described [82], consisting of a miniature coin-sized wearable sensor embedded with an IMU. The sensor is attached extraorally to the chin of the patient to enable mandibular motion sensing. Data on mandibular movement from the miniature IMU sensor are communicated to a dedicated smartphone application that automatically transfers them to a cloud-based infrastructure at the end of the recording. Data analysis is conducted using a dedicated machine learning algorithm that automatically scores sleep and respiratory events. Tracking mandibular motion with this wearable sensor has shown potential for the diagnosis of OSA [89,90,91] and relieving bruxism [174].

## 5. Research Impact

### 5.1. Intraoral Applications

Miniature wearable sensors can provide continuous real-time information through dynamic, non-invasive measurements and have been used occasionally used in orthodontic research. For the precise spatial control of tooth movement and to lessen adverse consequences such as root resorption, it is desirable to measure three-dimensional (3D) force–moment (F/M) systems utilized for correcting dental malposition. In the arena of intelligent orthodontic appliances based on smart brackets, 3D F/M systems can be measured with accuracy [175]. These smart brackets seek to quantitatively determine each of the six F/M components. Rues and co-investigators adopted a strategy predicated on the idea that F/M systems externally applied to the tooth can be recreated by accurately describing the mechanical stress profiles inside the bracket, which consists of a micro coil made by electroplating gold in a photoresist mask and a 0.35 m stress mapping chip. Twenty-four transistor-based stress sensors are strategically placed throughout the chip surface to measure the difference between in-plane normal stresses or in-plane shear stress. The technology laid the groundwork for the creation of smart brackets with dimensions of 2 × 2.5 × 0.73 mm^3^ and a power consumption of 1.75 mW.

A single-axis piezoresistive accelerometer (model 4374; Bruel & Kjaer, Norcross, GA, USA) attached to orthodontic brackets was used to investigate differences between passive and active ligation methods [176]. The study found significant differences in bracket-archwire frictional resistances with variations in the amplitude of archwire vibration. Medium (150 mV) and high (190 mV) amplitudes produced statistically significant reductions in the time required to overcome friction compared to low-amplitude values (110 mV).

A wireless low-power capacitive humidity sensor was described for the detection of orthodontic bond failure. The system comprises a wireless power transfer circuitry, a humidity sensor, and a rectifier. The humidity sensor consists of two branches of inverters with reference and sensing capacitance connected to their outputs, and changes in humidity levels lead to changes in the capacitance of the sensing element, creating a phase difference between the outputs of inverters [177].

A biochemical ligature (BraceIO) that changes color depending on saliva concentration (pH, nitric oxide, uric acid) was recently described [178]. The manufacturing process of the ligature, the external reading device, a technical evaluation of the absorption time, the colorimetric measurements, and the color map at the biosensor level of the app were described by the researchers. They retain the traditional ligature shape, portability, and aesthetics while incorporating biosensors. To access multiple biodata and create a seamless interactive device, the researchers also proposed a version that captures metabolic changes with different biosensor ligatures attached to each tooth.

The maintenance of oral maintenance is important during orthodontic treatment. A smart toothbrush that is connected to various sensors and synced to a smartphone-based application via Bluetooth was recently announced [179,180]. This toothbrush tracks brushing progress in real time, provides progress reports, and helps identify focus areas that need further brushing. However, further research is needed to maximize the reusability of these data and link the data to individual clinical records for use in oral hygiene management.

### 5.2. Extraoral Applications

Improving smile characteristics is a goal of orthodontic treatment. Smiles may be social/posed or authentic/spontaneous [181] with differences in the amount of contraction of the involved muscles as well as the timing and amplitude of these smiles [182,183,184]. Smile analysis is usually based on evaluating static relationships of orofacial structures [185] using two-dimensional photographs [186,187] as opposed to exploring dynamic characteristics [181,188,189]. The assessment of smile dynamics was made possible with the emergence of three-dimensional video imaging techniques [190,191,192]. However, data are based on very short videos, and further research has been recommended in this field [193].

Facial recognition is a relatively new area of research. Wearable interfaces that can help in recognition of facial expressions, including smiling, have been described in the literature [194,195,196,197]. Early attempts at real-time facial expression analysis were based on camera-based methods that were cumbersome, not discreet, and heavy to wear [194]. Although accurate, the initial prototypes needed several improvements. With miniaturization, camera modules have become small enough to wear comfortably and have been used in commercial wearable devices [198,199].

The Facial Action Coding System (FACS) is a standardized facial-movement-labeling system that removes subjective inferences [200], has been automated, and is undergoing continuous development [201,202]. The placement of electrodes on facial muscles can measure the facial EMG signals for quantification of facial expressions [203,204,205]. Wearable sEMG devices have been used to detect the frequency and duration of smiling episodes. The drawbacks of facial sEMG include the limitation of the number of sensors that can be used, and even the act of electrode placement overlying muscles of facial expression hinders facial expressions [206]. The use of electromyographic signals distal to the site of the active muscles for facial expression recognition is yet another approach that researchers have attempted in order to overcome some of these drawbacks. Distal EMG was implemented on a four-channel EMG wearable attached to the sides of the face at eye level, making it possible to reliably measure smiles in different situations without obstructing facial movement [207]. A mix of distal electromyographic and other biological signals was used by a novel unobtrusive wearable device that applied computational methods to read facial expressions [195]. Combining local facial expressions with other biosignals was found to yield better outcomes than single-channel devices [208]. A novel flexible epidermal electronic-sensor-based wearable system was recently described that utilizes high-fidelity sEMG signals and artificial intelligence algorithms to classify and recognize facial emotions [209].

A behind-the-ear wearable that measures EEG and electrooculography (EOG) data, in addition to EMG to objectively quantify facial muscle movement, was used in a pilot study to discriminate muscle activities such as chewing, talking, and swallowing [210]. Neck-mounted wearables (NeckFace) in two different form factors (necklace and neckband) were tested to continuously track full facial expressions in specific tasks but presented challenges for adoption in real-life settings [211]. Another approach is the use of motion capture, and advances have enabled the identification of posed and spontaneous smiling using a mixture of analyzing facial expressions as well as body language [212]. A head-mounted camera-based wearable system (VICON Cara helmet) was recently used to develop a normative database of 3D facial ranges of motion in adults [213]. Most of the existing research work describes early prototype wearable devices that have been tested in constrained or semi-constrained conditions with only few tracking the smile in unconstrained real-life settings.

## 6. Risk Assessment and Regulatory Aspects

Although orofacial wearable devices are widely considered “non-invasive,” potential risks of chemical exposure need to be closely examined as they are located in or near the oral cavity. When using an intraoral wearable, electronic components, including the printed circuit board and the sensor/electrode, are at risk of being exposed to the oral environment [214]. Even seemingly insignificant elements, such as the choice of solder for the circuit board, may be accidentally ingested, and it is important to consider the biocompatibility of all components used with wearables. Since wearable devices remain in close contact with patients’ bodies for extended periods of time, the risk of physical harm is also a concern. Additionally, allergic contact dermatitis to some components has been reported [215]. Furthermore, as wearables often remain in prolonged contact with the skin, extended use can cause bacterial build-up. Sweat accumulating at the skin-electrode interface of wearable devices can interfere with signal quality, especially with wet electrodes [216].

Wearable sensors enable multi-modal data collection in real time, raising new questions about data security and privacy, which act as barriers to their adoption. It would be difficult to develop the hardware and software systems associated with wearable sensors without keeping security and privacy in mind. The issues related to security and privacy of wearable technologies are mainly related to the compromise and modification of user data, unsecured communication, data theft, technologies with hidden components (cameras, microphones, etc.), lack of encryption, and other vulnerabilities. There are several opportunities for data leaks whenever a wearable device connects to a network. To protect the data of enterprises, a strong security system is necessary. Robust data security is essential for reliable, secure operation with wireless data transmission and smooth connectivity. The network connected to a user’s sensor data poses a risk of external and internal security attacks. Reliable authentication and encryption methods can reduce the risk of an external attack. The risks to data security increase when an internal attack involving people with authorized access to raw data occurs. For example, the attacker could insert falsified medical information, leading to disastrous consequences. The confidentiality, reliability, and integrity of health data generated by wearable biosensors are ensured by the early detection of such an intrusion and minimize security concerns.

Wearable orofacial sensors used for continuous real-time measurements generate a huge amount of data, requiring the use of cognitive data processing protocols [217]. Data cleaning and filtering techniques (such as noise filtering) are some methods commonly used to handle wearable data. These techniques can reduce wireless data transmission rates and energy consumption. Advanced data mining techniques can also develop relationships between sensor signals and clinical diagnoses in a large group of wearers and predict unpredictable events [218,219,220,221].

Wearable technology is an unregulated space, and most consumer wearable devices are not approved by the FDA and are therefore categorized as “wellness” products. Initiatives such as the Digital Health Software Precertification program [222] are steps in the right direction toward better regulation. To accelerate medical product innovation and development in the US context, the FDA has released a framework to implement its real-world evidence program to support the use of real-world data and support making regulatory decisions [223]. The legal data-protection regime in the United States also contains rules specific to health information, most notably the Health Insurance Portability and Accountability Act (HIPAA) of 1996. Section 264 of the act instructs the Secretary of Health and Human Services (HHS) to create recommendations on how to protect the privacy of individuals’ personal health information. The European General Data Protection Regulation (GDPR) is the EU’s general framework for processing the personal data of anyone and any processor in the EU. The GDPR sets out the rules for how personal data must be handled, which applies to a variety of software-controlled devices [224,225]. The real-world evidence created by real-world data can be used by manufacturers to support the design of clinical trials for innovative treatment approaches.

## 7. Futurology

Wearables empower individuals to better take care of their health [226], and demand for their use is anticipated to keep growing exponentially in the coming years [5]. With orthodontists being keen on adopting technology into their practices [227], the future for orthodontic applications of wearable devices looks promising. In the past, the direct interfacing of sensors onto tooth surfaces for oral health monitoring has been successful [228,229,230], and in the near future, bio-integrated and implanted biosensors for diagnosis, monitoring, treatment, and oral health management may soon be a reality.

Future work towards the practical use of sensors in the oral cavity will include detailed validation studies with important evaluations of safety aspects such as biocompatibility, potential toxicity, sterilization, and operational stability for intraoral use. Effective device encapsulation (including supporting electronic interfaces and power supply), and the use of biocompatible materials to eliminate risks associated with salivary contact, especially the leaching of chemicals into surrounding areas, is essential to protect the functionality of electronic devices. Despite advances in material sciences, developments in the sensing side, especially material characteristics of electrodes used in biopotential acquisition, have been slow [216]. However, novel materials such as smart-tattoos (set of biosensors implanted under the skin) [231] and soft epidermal electrodes [209] to measure biopotentials look promising. Qualitative aspects such as user experiences with wearables [232] have also not been extensively investigated and are an area for further research. The optimum wearable of the future will be one that can be easily installed for continuous monitoring and has long-lasting battery and very power low consumption, while wirelessly reporting all health-related information without constraining its user in any way and providing real-time feedback.

## Figures and Tables

**Table 1:** Wearable technologies for monitoring compliance with orthodontic appliances.

	Monitoring Mechanism	Orthodontic Appliance	Study
Extra-oral	Aledyne timer	Cervical pull headgear	Clemmer et al. 1979 [33]
	Commercial wrist-watch	Cervical pull headgear	Cureton et al. 1993 [39]
	Compliance science system	Cervical pull headgear	Brandao et al. 2006 [74]
	Thermochron i-Button	Cervical pull headgear	Bos et al. 2007 [75]
	TheraMon-microsensors	Facemask (forehead rest)	Arreghini et al. 2017 [52]
		HG and functional appliance	Arponen et al. 2020 [76]
Intra-oral	Thermochron i-Button	Oral appliances for sleep apnea	Inoko et al. 2009 [61]
	TheraMon-microsensors	Removable appliances	Schott et al. 2011 [44]
		Oral appliance for sleep-disordered breathing	Vanderveken et al. 2012 [49]
		Removable appliances/retainers	Pauls et al. 2013 [54]
		Hawley retainers/functional appliance retainers	Schott et al. 2013 [56]
		Removable maxillary expanders	Schott et al. 2014 [53]
		Active/passive orthodontic removable appliances	Tsomos et al. 2014 [60]
		Mandibular advancement device	Dieltjens et al. 2015 [50]
		Functional/active removable appliances	Schäfer et al. 2015 [55]
		Functional appliances	Arreghini et al. 2017 [52]
		Expansion plates, functional appliances, and retention plates.	Schott et al. 2017 [59]
		Hawley and vacuum-formed retainers	Vagdouti et al. 2019 [58]
		Twin Block	Frilund et al. 2022 [45]
	SMART Microsensor	Maxillary Hawley retainer	Hyun et al. 2015 [57]
	DentiTrac thermal sensor	Mandibular advancement device	Gjerde et al. 2018 [47]
	Food-grade dye	Clear aligners	Tuncay et al. 2009 [72]

## Data Availability

Not applicable.
